# An International Comparison of the Community Mobility Patterns of Older Adults

**DOI:** 10.1093/geroni/igaa057.2335

**Published:** 2020-12-16

**Authors:** Lizette Swanepoel, Isabelle Gelinas, Barbara Mazer

**Affiliations:** 1 Stellenbosch University, Cape Town, Western Cape, South Africa; 2 McGill University, Montreal, Quebec, Canada; 3 McGill University School of Physical and Occupational Therapy, Montreal, Quebec, Canada

## Abstract

Community mobility in older adults is important for maintaining health, quality of life and social participation. Globally, older adults who are non-drivers, access their community through various modes of transport to maintain community mobility. This international cross-sectional cohort study (n=246) explored the mobility patterns of older adults and examined their access to out-of-home activities and health related quality of life in seven countries. Quality of life was determined using EQ-5D-5L and was generally high among all participants. Findings from the study indicate that a complex myriad of factors influence safe transport mobility in older adults. Results suggested that inclement weather and place of residence negatively impacted access to out-of-home activities, yet these factors but did not increase use of public transport. Given the complexity of transportation use and mobility patterns in older adults, an individualised approach may be necessary to keep older adults connected to their out-of-home activities.

